# Supplementary material for: Complexome profiling of the Chlamydomonas psb28 mutant reveals TEF5 as an early PSII assembly factor
Source: Plant Cell. 2025 Mar 17;37(6):koaf055. doi: 10.1093/plcell/koaf055 (PMC12164590; doi:10.1093/plcell/koaf055)
Supplement: koaf055_Supplementary_Data [file koaf055_supplementary_data.zip › psb28_interactive.zip!/plots/intensity/qualityPlots/peptides/_Cre06.g263250.t1.1.html]

### Cre06.g263250.t1.1

Trivial name:   
SeqCoverage [median]: 73.10  
Mass: 322.93 kDa  
  
 

| Sequence | MissedCleavage | Mass [Da] | wtIntensity | muIntensity | Score |
| --- | --- | --- | --- | --- | --- |
| AAATGAAAAAGSQLASAQEAGK | 0 | 1871.93 | 362523.73 | 361640.90 | 282.86 |
| AAATGVAAAAGSQLASAQEAGK | 0 | 1899.96 | 2511.36 | 4136.04 | 145.52 |
| AAVADATGAASSAAADAK | 0 | 1517.73 | 4198.87 | 9125.23 | 153.32 |
| AAVADATGAASSAAADAR | 0 | 1545.74 | 121818.06 | 195621.42 | 321.86 |
| AAVADATGAASSAAADTR | 0 | 1575.75 | 14734.23 | 21134.20 | 154.51 |
| AAVADATGAASSAATDAK | 0 | 1547.74 | 2561.11 | 3313.55 | 109.04 |
| AAVADATGAASSAATDAR | 0 | 1575.75 | 18796.72 | 32180.75 | 148.41 |
| AAVADVSGLASSAAAGAK | 0 | 1515.79 | 702.49 | 2285.73 | 91.62 |
| AAVTDATGAASSAAADAR | 0 | 1575.75 | 53731.12 | 85332.92 | 149.19 |
| AAVTDATGAASSAAADTR | 0 | 1605.76 | 13233.48 | 8645.76 | 117.18 |
| AAVTDATGAASSAATDAR | 0 | 1605.76 | 11878.59 | 13480.85 | 135.58 |
| AAVVDATGAASSAAADAR | 0 | 1573.77 | 15755.45 | 25914.26 | 101.05 |
| AAVVDATGAASSAAADTR | 0 | 1603.78 | 1901.36 | 2841.77 | 280.91 |
| ATTSGAVSAAASK | 0 | 1120.57 | 672.97 | 782.03 | 99.57 |
| ATVAGALTGAAAAAADAK | 0 | 1499.79 | 981.17 | 2789.44 | 99.04 |
| GAAESTASAASGALGTQTGTLQEQTK | 0 | 2435.18 | 427.73 | 1347.25 | 54.14 |
| SAQDGAAGAAATAR | 0 | 1216.58 | 832.81 | 911.77 | 104.24 |
| SVAAVAASATTAGEGAVLK | 0 | 1672.90 | 705.44 | 1481.70 | 131.53 |
|  |  |  | 627966.7 | 772965.6 |  |
